# Sustainable Sources of Biomass for Bioremediation of Heavy Metals in Waste Water Derived from Coal-Fired Power Generation

**DOI:** 10.1371/journal.pone.0036470

**Published:** 2012-05-09

**Authors:** Richard J. Saunders, Nicholas A. Paul, Yi Hu, Rocky de Nys

**Affiliations:** 1 School of Marine and Tropical Biology & Centre for Sustainable Tropical Fisheries and Aquaculture, James Cook University, Townsville, Australia; 2 Advanced Analytical Centre, James Cook University, Townsville, Australia; Lawrence Berkeley National Laboratory, United States of America

## Abstract

Biosorption of heavy metals using dried algal biomass has been extensively described but rarely implemented. We contend this is because available algal biomass is a valuable product with a ready market. Therefore, we considered an alternative and practical approach to algal bioremediation in which algae were cultured directly in the waste water stream. We cultured three species of algae with and without nutrient addition in water that was contaminated with heavy metals from an Ash Dam associated with coal-fired power generation and tested metal uptake and bioremediation potential. All species achieved high concentrations of heavy metals (to 8% dry mass). Two key elements, V and As, reached concentrations in the biomass of 1543 mg.kg^−1^ DW and 137 mg.kg^−1^ DW. Growth rates were reduced by more than half in neat Ash Dam water than when nutrients were supplied in excess. Growth rate and bioconcentration were positively correlated for most elements, but some elements (e.g. Cd, Zn) were concentrated more when growth rates were lower, indicating the potential to tailor bioremediation depending on the pollutant. The cosmopolitan nature of the macroalgae studied, and their ability to grow and concentrate a suite of heavy metals from industrial wastes, highlights a clear benefit in the practical application of waste water bioremediation.

## Introduction

The use of algae to remove pollutants from water, algal bioremediation, has been well studied over the past 40 years [1], [2], [3], [4]. Since the 1980s considerable research effort has been devoted to the development of algal biosorbents to remediate pollutants, particularly heavy metals [5]. At the laboratory scale these preparations have proven spectacularly successful at sorbing pollutants, especially heavy metals [5], [6]. However, uptake of the concept has been lack-lustre, evidenced by the lack of successful commercialisation (e.g. AlgaSORB circa 1991). This is likely because available algal (seaweed) biomass that is produced has established markets as food and as food ingredients (see Chopin and Sawhney [7] for market details). Furthermore, amongst the most successful preparations developed are those from brown macroalgae [8] which already have particularly well established markets and command a high price. A cheaper, reliable and locally derived source of biomass is critical [4], and remains a bottleneck for commercial applications of algae in bioremediation.

Bioconcentration, defined as the accumulation of a substance from the environment by the live algal biomass, offers an alternative approach to biosorption, defined as adsorption of metal ions on dead biomass [6]. We have used the term bioconcentration rather than bioaccumulation which is often associated with the process of trophic level transfer, and thus can be confused with biomagnification of pollutants [9], [10]. While there has been substantial research into algal biosorption, there has been remarkably little research devoted to algal bioconcentration for heavy metal bioremediation, however see Sternberg and Dorn [11]. The common justifications for researching algal biosorption are that the biomass is inexpensive [5] and has greater binding capacity than live biomass [12], [13]. However, biosorption approaches rely largely on specific binding of elements to active sites on cell walls [6] whereas bioconcentration may occur in numerous cellular structures or compartments, e.g. vacuolar accumulation of heavy metals [14] and can occur simultaneously for metals in different ionic states, cf. anionic or cationic arsenic: Ghimire et al. [15]. The key factors for bioconcentration to be successful are the ability of the algae to target numerous heavy metals [16], [17] and the capacity to grow and survive in the waste water stream. Thus, bioremediation with living biomass is a combination of both bioconcentration and biomass productivity, as high growth rates will provide new cellular material to bind and capture metals. The process is complicated when different growth states or age of algal tissue influence the selectivity and concentrations of specific metals [17]. In these cases factors that affect growth may also impact capacity for bioconcentration, making it essential to simultaneously quantify bioconcentration and algal growth in the relevant waste water stream.

**Table 1:** Elemental composition (mg.L^−^1) of the different water sources used in the growth and elemental uptake experiment. < indicates that concentration was below the limits of detection.

Element	Ash Dam	Ash Dam +f/2	Town supply+ f/2
Aluminium	0.08	0.06	<0.01
Arsenic	0.0175	0.017	<0.001
Boron	2.26	2.28	<0.05
Cadmium	0.0004	0.00035	<0.0001
Calcium	197.0	189.5	10.0
Chromium	<0.001	0.001	<0.001
Copper	0.004	0.0185	0.0215
Iron	0.275	1.55	0.7
Lead	<0.001	<0.001	<0.001
Magnesium	69.5	60.5	2.0
Manganese	0.002	0.0775	0.103
Mercury	<0.0001	<0.0001	<0.0001
Molybdenum	0.8595	0.9345	0.017
Nickel	0.016	0.026	<0.001
Phosphorous	<1.0	1.0	1.0
Potassium	30	31.5	5.5
Selenium	0.06	0.02	<0.01
Sodium	335.5	332	34.5
Strontium	1.365	1.43	0.05
Vanadium	0.565	0.6	<0.01
Zinc	0.231	0.3585	0.16

**Figure 1:**
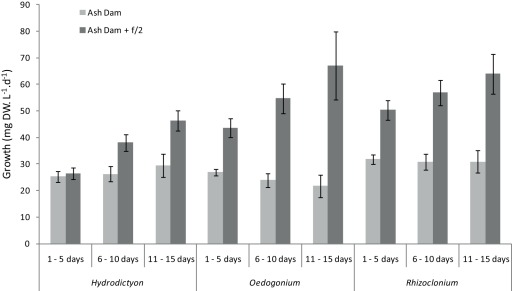
Growth of algae cultured in Ash Dam water and Ash Dam water with the addition of f/2 media over a fifteen day period. All three species of algae had higher growth rates with the addition of f/2 and there was also a significant influence of time on growth for all species. Error bars are standard error.

Living macroalgae play an important role in pollution management [18]. The earliest uses of algae in pollution control were developed for sewage waste water where their uptake of N and P was harnessed [1]. The capacity for some algae for luxury uptake or bioconcentration of N and P has also been utilised for bioremediation of waste water from aquaculture (integrated aquaculture) [19]. More recently, the role of carbon in human induced climate change has resulted in a considerable focus on carbon capture and storage (CCS). One approach that has attracted considerable commercial interest is the culture of algal biomass to sequester or recycle carbon (BioCCSR) [20]. The success of these applications of algal bioremediation processes lies in the fact that the biomass is cultured in situ and does not require wild harvested biomass. In our study, we have applied this concept and considered the culture of algae in polluted water as a remediation strategy, circumventing the need to source biomass for remediation and providing a continuous management strategy for heavy metal extraction. The use of macroalgae rather than microalgae also negates the difficulties associated with harvesting biomass. The culture of live biomass also has the additional value-adding potential for BioCCSR as well as by-product development [21]. Such by-products depend entirely on the waste water streams that are to be remediated.

**Table 2:** Average concentration (mg.kg^−1^) of elements ± s.e. for each alga, treatment and control.

Element	Hydrodictyon			Oedogonium			Rhizoclonium		
	Ash Dam*	Ash Dam +F2	Stock cultures	Ash Dam*	Ash Dam +F2	Stock cultures	Ash Dam	Ash Dam +F2	Stock cultures
Aluminium	1360.00 ± 280.00	879.67±145.59	24.75±3.15	620.50±231.50	1222.33±127.94	19.40±4.84	627.67±221.35	978.00±9.17	29.95±3.35
Arsenic	44.45±2.45	80.80±6.39	1.68±0.14	54.55±21.85	137.00±4.58		50.23±13.83	105.57±5.01	
Boron	78.75±16.45	247.33±38.75	14.27±2.44	511.40±471.60	414.00±24.34	4.03±0.81	310.67±54.04	533.00±4.73	6.37±0.84
Calcium	8455.00±2045.00	51733.33±6227.18	3320.00±369.50	8250.00±2750.00	87433.33±7411.78	3226.67±67.41	17633.33±3331.83	66933.33±726.48	2660.00±193.13
Cadmium	8.05±0.16	0.23±0.03		4.65±1.62	0.54±0.10		3.50±0.92	0.32±0.04	
Chromium	5.40±1.12	5.29±0.42	2.03±0.19	5.28±1.12	3.90±0.40	0.82±0.03	4.20±1.41	6.38±0.41	1.34±0.14
Copper	42.85±1.65	42.07±0.71	12.80±1.42	14.35±5.06	85.13±6.51	9.43±0.27	14.47±7.42	69.10±7.36	13.07±0.72
Iron	3770.00±530.00	20366.67±976.96	4893.33±832.51	4010.00±1850.00	24933.33±536.45	1108.33±338.65	3736.67±2123.41	24633.33±726.48	1206.67±184.15
Mercury									
Potassium			31200.00±1289.70	30500.00±10700	8440.00±832.83	27900.00±115.47	33450.00±8858.99	20433.33±3307.74	26133.33±1105.04
Magnesium	7225.00±155.00	23466.67±3433.82	3306.67±321.68	7490.00±2810.00	46566.67±3099.64	4523.33±100.39	6430.00±1374.37	36066.67±2682.25	2376.67±48.42
Manganese	357.50±44.50	986.00±127.99	697.00±144.51	208.00±91.00	1500.00±41.63	103.57±11.77	405.67±66.47	1493.33±109.14	210.00±14.84
Molybdenum	5.69±0.41	3.24±0.59	5.51±0.05		11.23±0.47	0.32±0.03	51.60±11.74	26.33±0.98	6.70±0.83
Sodium	2250.00±100.00	1390.00±85.44	953.50±23.50		2916.67±166.97		2686.67±483.75	3020.00±202.98	1306.67±138.60
Nickel	103.20±5.80	4.08±0.66		51.45±15.75	2.53±1.25		84.47±21.28	3.28±0.94	
Phosphorous	7475.00±1765.00	40333.33±4003.89	6260.00±485.83	8195.00±4005.00	66866.67±2772.08	6266.67±107.13	6866.67±1868.49	52200.00±1059.87	7516.67±180.95
Lead	2.93±0.08	1.10±0.29	1.57±0.31	2.44±0.33	1.21±0.30	0.43±0.12	2.85±1.54	1.23±0.20	0.60±0.02
Selenium	6.94±0.56	10.68±1.43		5.08±3.05	21.87±1.83		5.52±2.20	17.70±0.29	
Strontium	74.45±19.55	402.00±53.82	45.27±9.07	96.50±34.50	680.67±53.36	27.43±0.43	136.07±26.57	526.67±12.60	69.63±1.41
Vanadium	454.50±22.50	832.00±48.23	2.56±0.36	749.00±240.00	1543.33±46.67	0.53±0.04	326.00±147.76	1206.67±28.48	1.15±0.05
Zinc	4355.00±415.00	699.00±49.51	36.17±4.53	2320.00±720.00	1436.67±85.70	28.57±0.95	2003.33±529.22	960.33±151.99	30.47±1.07

* n = 2, all other cases n = 3, Empty cells are where concentration was below detection limits.

**Figure 2:**
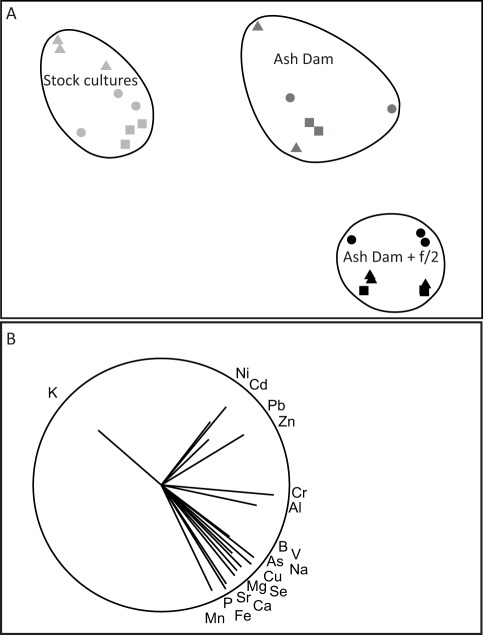
Non metric multidimensional scaling plot showing the similarity between algal species and treatments based on elemental composition. (A) nMDS plot (Stress  = 0.05) with the groups from the cluster analysis superimposed. Triangles represent *Rhizoclonium*, circles represent *Oedongonium* and squares represent *Hydrodictyon*. (B) The same nMDS as A, with vectors superimposed, the length and direction of which indicates the strength of the correlation and direction of change between the two nMDS axes. Only elements with a correlation coefficient of 0.5 or greater are shown.

Coal fired power stations produce large volumes of polluted waste water when the ash collected in the flue, and that remaining in the furnace after the combustion of coal, is washed out. The contaminants in this water vary depending on the source of the coal but commonly include high concentrations of As, V, Mo and Se [22], [23], [24]. This presents a significant problem for industry as the water is often contaminated to such a degree that it must be stored and/or treated, at considerable cost. Storage dams containing large volumes of contaminated water are often associated with coal fired power stations and these ash storages come with environmental and human health risks [24]. Previous research on the use of algae for bioremediation has usually focussed on only one or two problematic elements, particularly Cd [6], [25]. However, most waste water streams including water contained in Ash Dams are complex and contain numerous hazardous elements [23], [24]. Therefore, a broad approach that considers uptake across a great number of elements is optimal in terms of bioremediation potential. In this study we investigated the potential of freshwater green algae to bioconcentrate a wide variety of elements in water sourced from an Ash Dam associated with the Tarong coal fired power station in south-eastern Queensland. Furthermore, we develop baseline data to establish a model for the bioremediation of Ash Dam water, and metals removal and recovery.

## Methods

### General

Three species of freshwater green macroalgae were utilised to investigate growth performance and to determine the bioconcentration factors for a variety of elements when grown in Ash Dam water sourced from the Tarong coal fired power station in south-eastern Queensland. The Tarong Power Station has a total generating capacity of 1400 megawatts and is amongst the largest power stations in Queensland, Australia.

**Table 3:** Elements grouped according to relative concentrations across all treatments and control (above).

Relative concentration across species between treatments and control	Elements
Stock culture < Ash Dam < Ash Dam+f/2	As, B, Ca, Cu, Mg, Se, Sr, V
(Stock culture ≈ Ash Dam +f/2)<Ash Dam	Cd, Ni, Pb, Zn
(Stock culture ≈ Ash Dam)<Ash Dam+f/2	Fe, P
(Stock culture ≈ Ash Dam)>Ash Dam +f/2	K
Stock culture < (Ash Dam ≈ Ash Dam+f/2)	Cr
Variation in pattern between species and treatment	Al, Mo, Na, Mn

Relative bioconcentration factors across species between treatments	Elements
Ash Dam <Ash Dam +f/2	As, B, Ca, Mg, P, Se, Sr, V
Ash Dam +f/2 < Ash Dam	Cd, Mn, Ni, Zn
Variation in pattern between species and treatment	Al, Cr, Cu, Fe, K, Mo

Elements grouped according to relative bioconcentration factors (see Table S 1 for full BCF results) between the Ash Dam and the Ash Dam with f/2 (below).

**Figure 3:**
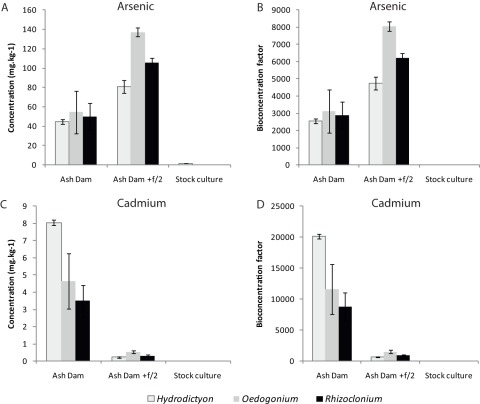
Examples of patterns of concentration and bioconcentration of metals in algae when cultured in Ash Dam water, Ash Dam water with f/2 and the stock cultures. (A) Concentration of arsenic. (B). Bioconcentration of arsenic. (C) Concentration of cadmium. (D) Bioconcentration of cadmium.

### Algae collections

The three species of green macroalgae were collected from aquaculture ponds and irrigation channels in Queensland. These were *Hydrodictyon* sp., *Oedogonium* sp., and *Rhizoclonium* sp. Species were identified to genus level using taxonomic keys [26] as each lacked defining characteristics to allow for identification to species level. The three freshwater algae are cosmopolitan genera from freshwater systems and are therefore representative of the macroalgae available in many freshwater environments. Furthermore, all can have rapid growth, particularly under eutrophic conditions, and are pest species in these environments [27], [28], [29], [30], [31], [32]. These algae range from the unbranched filamentous *Oedogonium* (cell diameter >2 µm) and *Rhizoclonium* (cell diameter >10–50 µm) to the net-forming *Hydrodictyon* (water net). Stock cultures of all algae were maintained in standard f/2 media [33]in the aquaculture facility at James Cook University prior to the experimental testing of growth and elemental uptake.

### Culture methods

The three algal species, *Hydrodictyon* sp., *Oedogonium* sp. and *Rhizoclonium* sp., were cultured in two treatments. The first was neat Ash Dam water, to determine growth potential without nutrient supplementation. The second treatment was an f/2 medium [33] in which freshwater was substituted for Ash Dam water (Ash Dam +f/2). This treatment was to determine growth and remediation potential of the algae in Ash Dam water under conditions where N, P and essential trace elements were not limiting. Ash dam water, with and without f/2 nutrients, had an initial pH of 7.0. The algae were cultured in 1.0 L Schott bottles at a stocking density of 1.0 g.L^−1^. The Schott bottles were placed randomly in a Sanyo Versatile Environmental Test Chamber (MLR-351). Mean light levels within the cabinet were 88 µmol photons.m^−2^.s^−1^ with a photoperiod of 12L: 12D and temperature was maintained at a constant 24°C. A complete water change was done at 10:00 am each day and the Schott bottles were rotated within the cabinet each day to avoid light bias. Every five days for a total of 15 days the replicates were dried using paper towel and weighed to the nearest 0.1 g. A stocking density of 1.0 g.L^−1^ was re-established at this time by increasing the volume of water. At the end of the 15 day culture period, the algae were harvested, patted dry on paper towel and then dried in a dehydrator for 48 hours at 45°C. The dry biomass was then weighed to the nearest 0.01 g. Dry matter content (DM) was calculated for each species and treatment at the end of the experiment (DM  =  dry weight/wet weight). All the dried biomass was stored in snap lock plastic bags at 4.0°C until it was analysed for elemental composition.

### Growth

Growth rates for each combination of treatment (Ash Dam and Ash Dam +f/2) and species were calculated using the fresh weight determined for three consecutive growth periods at day 5, 10 and 15 of the experiment. Growth rate (GR) was calculated using the equation GR  =  (*M_f_*−*M_i_*)/15*(DM) where, *M_f_*  =  fresh mass at day 15 and *M_i_*  =  initial mass. Mean growth rate (mg DW.L^−1^.d^−1^) are presented for each species x treatment combination (±1 standard error, from the three growth periods).

### Elemental analysis (algae)

The concentrations of 21 different elements, listed in Table 1, were determined for the algae grown in the two treatments (Ash Dam water and Ash Dam water with f/2) and unexposed biomass from the original stock that was maintained in dechlorinated water with f/2 media, henceforth referred to as stock cultures. All biomass was prepared for the analysis by drying in a dehydrator for 48 hours at 45°C. A minimum of 100 mg dry weight of algae was required for accurate determination of the elemental composition (see below). Three replicates were available in the majority of cases but in a few samples were pooled to provide duplicates.

For the elemental analyses, 100 mg samples of the dried algae were placed into digestion vessels with 2.5 mL SupraPure (Merck Germany) double distilled HNO_3_ and 1.0 mL AR Grade H_2_O_2_. The mixture was left to stand in the fume-hood for two hours to allow the reaction to complete. The vessels were then heated to 180°C in a microwave oven (Milestone Starter D) and maintained at this temperature for ten minutes. After cooling to room temperature, the digested samples were diluted to 100 mL with Milli-Q water in a volumetric flask. No further dilution was needed before elemental analysis.

Sample analysis was carried out using two instruments. Major elements (Al, Ca, K, Na and P) were measured using a Varian Liberty Series II Inductively Coupled Plasma Optical Emission Spectrometer (Melbourne, Australia). The remaining elements were measured using a Varian 820-MS Inductively Coupled Plasma Mass Spectrometer (ICP-MS) (Melbourne, Australia). External calibration strategy was used for both instruments with a series of multi-element standard solution containing all the elements of interest and the results were reported after subtracting the procedure blanks. Algae may be subject to Cl^-^ polyatomic ion interference, thus elements such as V, As, Se are susceptible to false positives. To assess this, one algal sample was spiked with 1 ppb As, Se and V and measured three times for quality control with recovery between 102 and 108% indicating no significant interferences. These analyses were done by the Advanced Analytical Centre (AAC) at James Cook University (JCU).

### Elemental analysis (water)

The three water sources in which the algae were cultured were analysed for the concentration of the same 21 elements as the algae (Table 1). These were Ash Dam water, the Ash Dam water with the addition of f/2 nutrients and stock culture water. The stock culture water was Townsville city supply that had been dechlorinated using a charcoal filter and supplemented with f/2 nutrients. Two replicate water samples of 200 mL each were taken from these three separate water sources. The samples were collected using a 200 mL syringe and passed through a Minisart 0.45 µm filter to remove particulates. The elemental measurements were done according to the USEPA 6020 ICP-MS standard following an acid digest. These measurements were done by the Australian Centre for Tropical Freshwater Research (ACTFR).

### Bioconcentration factor

Bioconcentration factor (BCF) is the ratio of the chemical concentration in the organism to the water [34]. BCF was calculated using the equation *BCF*  = *C_b_*/*C_w_* where, *C_b_* =  concentration of elements in the dry algal biomass (mg.kg^−1^) and *C_w_* =  concentration of elements in the water (mg.L^−1^).

### Statistical analysis

Growth rates were compared between treatment (Ash Dam and Ash Dam +f/2) and species (n = 3) by analysis of variance (ANOVA) with time (three, five day growth periods) as a blocked (random) factor in the model. Growth rate was log transformed to meet the assumptions of ANOVA. Mean Square (MS) error terms in the mixed model ANOVA were adjusted for calculation of F-ratios for treatment (MS treatment x time), species (MS species x time) and treatment x species (MS treatment x species x time). Multivariate statistics were used to determine if there were differences in elemental composition between species and algal biomass cultured in neat Ash Dam water, Ash Dam water +f/2 and stock culture biomass that was never exposed to ash dam water. A similarity matrix was calculated from the 4^th^ root transformed concentrations of all the different elements and a hierarchical agglomerative cluster analysis was done and superimposed on an nMDS. The 2D plots of the similarity matrix illustrate the clustering of the different treatments and show the direction and strength of change in the elemental composition of the algae.

## Results and Discussion

### Water analysis

The elemental composition of the Ash Dam water was complex and contained several heavy metals, such as V and As, at high concentrations (Table 1). The addition of f/2 nutrients to the ash water marginally increased the concentration of the essential elements Cu, Mn, Mo, Sr and Zn. The addition of f/2 nutrients increased Fe concentration by nearly 1.0 mg.L^−1^. The majority of heavy metals were undetectable in the stock culture water (dechlorinated town supply with f/2), although the f/2 nutrients provided low concentrations of some essential elements such as Fe, Cu, Sr, Mo, Mn and Zn (Table 1). The growth medium f/2 also supplied some nutrient detectable by the ICP- MS analysis as P.

### Growth and metal uptake

All three algae grew in the neat Ash Dam water and in Ash Dam water +f/2 nutrients. *Oedogonium* and *Rhizoclonium* had substantially higher growth rates with the addition of f/2 whereas *Hydrodictyon* grew marginally more with the addition of f/2 (Figure 1; ANOVA, species x treatment F_2,4_ = 20.04, p = 0.008). There was a subtle but significant influence of time on growth for all species with and without nutrient-addition, with a trend for increased growth in Ash Dam with f/2 over the three consecutive growth periods (Figure 1; ANOVA, treatment x time F_2,54_ = 7.91, p = 0.001). Growth was stable in the Ash Dam water from the first week 27.98 mg DW.L^−1^.d^−1^ (±1.16 SE) through to the third week 27.37 mg DW.L^−1^.d^−1^ (±2.29 SE). By contrast each species increased growth rate over the same period by ∼50% with the addition of f/2 to the Ash Dam water, on average from 40.12 mg DW.L^−1^.d^−1^ (±3.36 SE) to 59.14 mg DW.L^−1^.d^−1^ (± 4.84 SE). The addition of f/2 nutrients aided in the acclimatisation of the algae, evidenced by their increasing growth rate over the three week period of the experiment compared to the neat Ash Dam water, in which growth was stable over the period (Figure 1). Growth rates in the treatments were broadly comparable to those for all three species across a range of environments [27], [28], [29], [30], [31], [35].

The average concentration for the 21 elements across all species after exposure for 15 days is reported in Table 2. The stock cultures had the lowest concentration of all metals. The pattern of concentration of elements was more similar between algae, than across treatments, indicating that the water in which the algae were cultured significantly influenced their elemental composition. The multivariate analysis indicated that the stock cultures were uniformly low in heavy metal concentration compared to the Ash Dam and Ash Dam +f/2 treatments and that the addition of f/2 had a strong impact on the elemental composition of the algae cultured in the Ash Dam water (Figure 2).

Many elements showed consistent patterns of concentration across species and treatments. These could be categorised into several groups (Table 3). The most common pattern among treatments and species was that the concentration in the algal biomass of the element was Stock cultures<Ash Dam<Ash Dam+f/2. This pattern held true for the majority of elements including As, B, Ca, Cu, Mg, Se, Sr and V (see example of As, Figure 3a,b). The addition of f/2 media provided better conditions for uptake of these elements, presumably through increased metabolic rate. This group was identified when the vectors indicating the strength and direction of correlation on the nMDS plot were considered. These vectors indicated a greater concentration of this group of elements in the biomass cultured in the Ash Dam+f/2 water over that cultured in neat Ash Dam water (Figure 2a,b).

The second most common pattern, that occurred was [Stock cultures≈ Ash Dam +f2] <Ash Dam for Cd, Ni, Pb and Zn (see example of Cd, Figure 3c,d). These elements appear to be excluded when growth is increased by the addition of f/2 media. This group of elements was also defined clearly in the nMDS where the vectors for Cd, Ni, Pb and Zn indicated a similar strength and direction of change (Figure 2b). Thus, to maximise bioremediation of these elements it would be important to choose a compromise between algal growth promoted with f/2 media, and bioconcentration, which is substantially higher under sub-optimal growth conditions. Elements that were at very low concentrations in the water remained so in the algae. For example, Hg was undetectable in the water (Table 1) and was also undetectable in the algae (Table 3). However, Pb, which was undetectable in any of the water sources was found, albeit at low concentrations (<2 mg.kg^−1^), in the algae.

As bioconcentration factor is derived from the elemental composition of the algae it follows similar patterns. Bioconcentration was exhibited for all elements in at least one of the treatments and species with the exception of Na, which was always near equilibrium between the algal biomass and the water. BCF could not be calculated for Al, As, B, Cd, Ni, Se and V in the stock cultures because the concentration in the town water with f/2 nutrients was below detection limits (Table 1). However, none of these elements were concentrated to any significant extent in the stock cultures (Table 2). BCF could not be calculated for any algae for Hg or Pb because these were undetectable in any of the water sources. Given that the detection limits for Pb in the water were at least 0.001 mg.L^−1^ and concentration in the algae was over 1.0 mg.kg^−1^, a BCF of at least 1000 can be inferred. Extremely high bioconcentration factors of over 10,000 were observed for many elements including Al, Cu, Mn, Ni, P and Zn (Table S1). BCF for each alga was highest in the stock cultures treatments for Sr, Mg and K (Table S1). The same was true for Mo for all algae except *Oedogonium* which appeared to exclude, or at least limit uptake of Mo substantially. Considerable variation in BCF was exhibited between species and treatments for the elements Al, Fe, Cu and Ca (Table S1).

BCF was consistently highest for As, B, P, Se and V in the algae grown in Ash Dam water with f/2 media (Table 3 and Table S1; example for As Figure 3a,b). All algae also exhibited the fastest growth in this treatment, indicating that the rate of uptake of these elements is increased when growth rate increases. BCF was highest for Cd, Mn, Ni and Zn in all the algae grown in Ash Dam water without f/2 media (Table 3 and Table S1, example for Cd Figure 3c,d).

In this study we have not distinguished between adsorption and absorption but rather concentrated on the total metal concentration of the biomass as we were primarily concerned with total remediation potential of the biomass. Overall, growth of algae, either with, or without additional nutrients (f/2), demonstrated bioremediation of a very complex waste water stream. While the f/2 media enhanced algal growth it also resulted in markedly different but consistent patterns of bioconcentration across groups of elements. Two key elements in the Ash Dam water, As and V, were bioconcentrated to a higher level when additional nutrients (f/2) were provided. For these elements, the optimised bioremediation strategy would be to target optimised biomass productivities, e.g. through the supply of limiting nutrients. However, increased growth resulted in a concomitant reduction in the bioconcentration of Zn, Ni and Cd. This provides opportunities to tailor remediation strategies within targeted waste streams by controlling growth, through the provision of nitrogen in particular. Importantly, there is a trade off between growth and bioconcentration. If growth is sufficiently high, then there will be more biomass available for bioconcentration and thus the reduced rate of elemental uptake of Zn, Ni and Cd will have a negligible impact on overall bioremediation. These opposing patterns of bioconcentration could result from reactions with the f/2 media that force these specific elements into a form that is not readily bound to the algae, similar to other changes in water medium that influence ionic state and uptake [36]. These have not been measured. Alternatively, the altered metabolic state of the algae could impact the binding mechanisms on and within the cells [17]. Some of the observed differences may then be explained by changes in bioavailability [37], [38]. Future research should consider ways to provide nutrient that maximises metal bioavailability as well as growth.

### Comparison to biosorption

The majority of algal biosorption studies report metal binding capacity in the range of 10 to 100 mg.g^−1^ [5], [6], although some report even higher levels of biosorption, achieved by pretreatment of the biomass and manipulation of pH of the waste water [5]. In our study, individual metal concentration in the biomass rarely exceeded 5 mg.g^−1^ but total heavy metal load was comparable to the majority of biosorption studies, reaching over 60 mg.g^−1^ total heavy metals for all species cultured with f/2 medium. The high concentrations achieved in our study may be the result of the live algae bioconcentrating within the cell vacuole as well as binding to the cell walls. This provides additional sites for elemental storage in live algae [14], indeed, metals can even be bound in free sugars e.g. arseno-sugars for arsenic [39]. This, coupled with the ability of live biomass to grow and provide new substrate continuously, may make up for any short-fall in uptake capacity. Bioremediation may also be enhanced by manipulation of growing conditions of the algae. Integrating culture with other waste streams, such as municipal waste to provide N and P, and control of pH through the addition of CO_2_, from flue gas from the power station, could yield higher rates of bioremediation through increased biomass productivity in the supply of the growth limiting nutrient carbon e.g. Israel et al. [40]. Furthermore, the control of waste water pH (by manipulating the dissolved CO_2_ from coal-fired power station) offers additional opportunities to target bioconcentration of specific elements [36] or ionic states [41].

The justification for biosorption using algal biomass for heavy metal remediation often relies on costs, notably without comprehensive life cycle analyses that incorporate costs of processing and/or transport of biomass to and from polluted sites [5]. Using the elemental concentrations of problematic elements in the algae in this study (*Oedogonium* with V concentration at 1543 mg.kg^−1^ and *Rhizoclonium* with As concentration at 105 mg.kg^−1^) and realistic algal biomass yields of 20 g DW.m^−2^.d^−1^, which equates to 73 tonne.ha^−1^.annum^−1^ [42], a 100 ha culture area could remove one tonne of As per annum, providing significant removal of an environmentally sensitive element. In addition, such a culture would remove nearly 11 tonnes of V per annum as well as substantial removal of most other metals at the same time. However, estimating the costs of large scale algal culture is difficult [43] and estimates will vary widely by region. While algal culture may not be a panacea for heavy metal pollution, consideration of complementary remediation strategies where regional alliances between organic waste producers (N and P) and chemical waste producers (metals) are developed [5] will be an important step in developing practical, cost effective algal bioremediation.

## Supporting Information

Table S1 **Average BCF of elements ± s.e. for each alga, treatment and control.** * n = 2, all others n = 3, Empty cells are where data was not available as the concentration in the water and/or the alga was below detection limits.
(DOCX)
