# Supplementary material for: Sustainable Sources of Biomass for Bioremediation of Heavy Metals in Waste Water Derived from Coal-Fired Power Generation
Source: PLoS One. 2012 May 9;7(5):e36470. doi: 10.1371/journal.pone.0036470 (PMC3348934; doi:10.1371/journal.pone.0036470)
Supplement: Table S1 — Average BCF of elements ± s.e. for each alga, treatment and control. * n = 2, all others n = 3, Empty cells are where data was not available as the concentration in the water and/or the alga was below detection limits. (DOCX) [file pone.0036470.s001.docx]

Table S1. Average BCF of elements ± s.e. for each alga, treatment and control. * n=2, all others n=3, Empty cells are where data was not available as the concentration in the water and/or the alga was below detection limits.

| Element | *Hydrodictyon* | | | *Oedogonium* | | | *Rhizoclonium* | | |
| --- | --- | --- | --- | --- | --- | --- | --- | --- | --- |
|  | Ash Dam* | Ash Dam + f/2 | Control+f/2 | Ash Dam* | Ash Dam + f/2 | Control + f/2 | Ash Dam | Ash Dam + f/2 | Control + f/2 |
| Aluminium | 17000 ± 3500 | 14661 ± 2427 |  | 7756 ± 2894 | 20372 ± 2132 |  | 7846 ± 2767 | 16300 ± 153 |  |
| Arsenic | 2540 ± 140 | 4753 ± 376 |  | 3117 ± 1249 | 8059 ± 270 |  | 2870 ± 790 | 6210 ± 295 |  |
| Boron | 35 ± 7 | 109 ± 17 |  | 226 ± 209 | 182 ± 11 |  | 137 ± 24 | 234 ± 2 |  |
| Calcium | 43 ± 2 | 273 ± 2 | 332 ± 2 | 42 ± 2 | 461 ± 2 | 323 ± 0 | 90 ± 2 | 353 ± 0 | 266 ± 1 |
| Cadmium | 20125 ± 400 | 671 ± 96 |  | 11625 ± 4050 | 1530 ± 276 |  | 8742 ± 2290 | 906 ± 128 |  |
| Chromium |  | 5293 ± 416 |  |  | 3903 ± 400 |  |  | 6380 ± 411 |  |
| Copper | 10713 ± 4 | 2274 ± 1 | 595 ± 3 | 3586 ± 21 | 4602 ± 5 | 439 ± 1 | 3618 ± 31 | 3735 ± 7 | 608 ± 1 |
| Iron | 13709 ± 16 | 13140 ± 6 | 6991 ± 14 | 14581 ± 56 | 16086 ± 3 | 1583 ± 12 | 13588 ± 66 | 15893 ± 4 | 1724 ± 6 |
| Mercury |  |  |  |  |  |  |  |  |  |
| Potassium |  |  | 5673 ± 235 | 1017 ± 357 |  | 5073 ± 21 |  | 649 ± 105 | 4752 ± 201 |
| Magnesium | 104 ± 0 | 388 ± 3 | 1653 ± 4 | 108 ± 4 | 770 ± 2 | 2262 ± 1 | 93 ± 2 | 596 ± 2 | 1188 ± 1 |
| Manganese | 178750 ± 53 | 12723 ± 15 | 6767 ± 17 | 104000 ± 141 | 19355 ± 4 | 1006 ± 4 | 202833 ± 74 | 19269 ± 10 | 2039 ± 3 |
| Molybdenum | 7 ± 1 | 4 ± 1 | 324 ± 3 |  | 12 ± 1 |  | 60 ± 14 | 28 ± 1 | 394 ± 49 |
| Sodium | 7 ± 0 | 4 ± 0 | 18 ± 9 |  | 9 ± 1 |  | 8 ± 1 | 9 ± 1 | 40 ± 4 |
| Nickel | 6450 ± 363 |  |  | 3216 ± 984 | 97 ± 48 |  | 5279 ± 1330 | 126 ± 36 |  |
| Phosphorous |  | 40333 ± 4004 | 6260 ± 486 |  | 66867 ± 2772 | 6267 ± 107 |  | 52200 ± 1060 | 7517 ± 181 |
| Lead |  |  |  |  |  |  |  |  |  |
| Selenium | 116 ± 9 | 534 ± 71 |  | 85 ± 51 | 1093 ± 91 |  | 92 ± 37 | 885 ± 14 |  |
| Strontium | 55 ± 2 | 281 ± 2 | 905 ± 6 | 71 ± 3 | 476 ± 2 | 549 ± 0 | 100 ± 2 | 368 ± 1 | 1393 ± 1 |
| Vanadium | 804 ± 40 | 1387 ± 80 |  | 1326 ± 425 | 2572 ± 78 |  | 577 ± 262 | 2011 ± 47 |  |
| Zinc | 18853 ± 13 | 1950 ± 3 | 226 ± 2 | 10043 ± 31 | 4007 ± 4 | 179 ± 1 | 8672 ± 25 | 2679 ± 8 | 190 ± 1 |
